# Machine learning using clinical data at baseline predicts the efficacy of vedolizumab at week 22 in patients with ulcerative colitis

**DOI:** 10.1038/s41598-021-96019-x

**Published:** 2021-08-12

**Authors:** Jun Miyoshi, Tsubasa Maeda, Katsuyoshi Matsuoka, Daisuke Saito, Sawako Miyoshi, Minoru Matsuura, Susumu Okamoto, Satoshi Tamura, Tadakazu Hisamatsu

**Affiliations:** 1 Department of Gastroenterology and Hepatology, Kyorin University School of Medicine, 6-20-2 Shinkawa, Mitaka-shi, Tokyo, 181-8611 Japan; 2 Department of Electrical, Electronic and Computer Engineering, Faculty of Engineering, Gifu University, 1-1 Yanagido, Gifu-shi, Gifu, 501-1193 Japan; 3 Division of Gastroenterology and Hepatology, Department of Internal Medicine, Toho University Sakura Medical Center, 564-1 Simoshizu, Sakura-shi, Chiba, 285-0841 Japan; 4 Department of General Medicine, Kyorin University School of Medicine, 6-20-2 Shinkawa, Mitaka-shi, Tokyo, 181-8611 Japan

**Keywords:** Machine learning, Ulcerative colitis, Predictive markers

## Abstract

Predicting the response of patients with ulcerative colitis (UC) to a biologic such as vedolizumab (VDZ) before administration is an unmet need for optimizing individual patient treatment. We hypothesized that the machine-learning approach with daily clinical information can be a new, promising strategy for developing a drug-efficacy prediction tool. Random forest with grid search and cross-validation was employed in Cohort 1 to determine the contribution of clinical features at baseline (week 0) to steroid-free clinical remission (SFCR) with VDZ at week 22. Among 49 clinical features including sex, age, height, body weight, BMI, disease duration/phenotype, treatment history, clinical activity, endoscopic activity, and blood test items, the top eight features (partial Mayo score, MCH, BMI, BUN, concomitant use of AZA, lymphocyte fraction, height, and CRP) were selected for logistic regression to develop a prediction model for SFCR at week 22. In the validation using the external Cohort 2, the positive and negative predictive values of the prediction model were 54.5% and 92.3%, respectively. The prediction tool appeared useful for identifying patients with UC who would not achieve SFCR at week 22 during VDZ therapy. This study provides a proof-of-concept that machine learning using real-world data could permit personalized treatment for UC.

## Introduction

Ulcerative colitis (UC) is one of the major phenotypes of inflammatory bowel disease (IBD), and it is characterized by chronic colonic inflammation with periods of remission and relapse. Although the pathophysiology of ulcerative UC remains unclear, more patients has been able to achieve remission with the improvement of therapeutic options and strategies, which has led to better long-term prognosis^1–4^. At present, various molecular targeted drugs, such as calcineurin inhibitor [cyclosporine A and tacrolimus (TAC)], anti-tumor necrosis factor alpha (TNFα) antibodies [adalimumab (ADA), golimumab, and infliximab (IFX)], anti-α_4_β_7_ integrin antibody [vedolizumab (VDZ)], anti-IL12/23p40 antibody [ustekinumab (UST)], and Janus kinase (JAK) inhibitor [tofacitinib (TOF)], are particularly used for treating patients with steroid-dependent/refractory UC. Meanwhile, in most clinical settings, it is challenging for physicians to identify the most effective molecular targeted drug for individual patients. When a physician considers starting a molecular targeted medication, the patient must have active disease that requires additional therapeutic intervention, that is, appropriate selection of a medication without delay is expected. In general, there is no guide for selecting the most suitable molecular targeted drug for the individual patient at present. This lack of a guide affects both patient outcomes and medical costs. Molecular targeted drugs are far more expensive than conventional medications such as 5-aminosalicylic acid (5-ASA), immunomodulators [e.g., azathioprine (AZA)], and steroids. The use of ineffective molecular targeted medications can represent a socioeconomic burden. Thus, predicting the efficacy of a molecular targeted medication before administration is crucial in this molecular-targeted therapy era.

The real-world pooled outcome of VDZ demonstrated that the rates of clinical response and remission at week 14 were 51% and 31%, respectively^5^. Given these rates, other medications might be more effective than VDZ for some patients with UC, and the prediction of VDZ efficacy in advance could provide these patients with an opportunity to initially receive another therapy. Several studies investigated the predictors of response to VDZ in UC^6,7^. Among clinical factors at baseline, serum C-reactive protein (CRP) levels^8,9^, serum albumin concentrations^7^, the Mayo Clinic score^9^, previous exposure to anti-TNF agents^7,10^, disease duration^7^, and endoscopic activity^7^ have been reported to be associated with the clinical efficacy of VDZ in patients with UC. These previous studies employed statistical methods such as univariate and multivariate analyses to search for the predictors. In this study, we hypothesized that a new approach using machine learning could illuminate predictive factors of VDZ efficacy for UC that have not been detected as statistically significant using the conventional statistical approaches. In the present study, we investigated clinical features at baseline (week 0) that affect steroid-free clinical remission (SFCR) during VDZ therapy at week 22 and developed a prediction tool. Random forest (RF)^11^ is an ensemble learning algorithm generating decision trees based on the training data. RF can also estimate the relative importance score for each feature. That is, RF allows the analysis of many factors simultaneously and provides insights into the contribution of each factor to the eventual outcome (i.e., achievement vs. no achievement of SFCR at week 22). We employed this method for clinical data at week 0 for patients with UC who started VDZ treatment for the induction of remission (training cohort), and the extracted factors were used to develop a prediction tool. The predictive accuracy of the tool was evaluated with another data set of patients who received VDZ for UC (test cohort).

The merit of this study is attempting to establish a prediction model based on generally available clinical information that was collected in daily practice. This is crucial for applying a machine learning-based prediction tool to the clinical setting. This pioneering work provides a proof-of-concept that the machine-leaning approach can be a new strategy for investigating predictors of the treatment efficacy in patients with UC and developing a prediction tool.

## Methods

### Study subjects

We retrospectively collected clinical data at baseline (week 0) and examined the clinical activity of UC at week 22 in 34 patients who (1) started VDZ at Kyorin University Hospital between September 2019 and April 2020 for the induction of remission, (2) underwent blood testing at week 0, and (3) underwent examination at Kyorin University Hospital at week 22 (training cohort, Cohort 1). As an extra-facility cohort, 35 patients with UC at Toho University Sakura Medical Center who (1) started VDZ between January 2019 and June 2020 for the induction of remission, (2) underwent blood testing at week 0, and (3) underwent examination at Toho University Sakura Medical Center at week 22 were analyzed (Cohort 2). The diagnosis of UC was confirmed using the clinical practice guidelines for IBD of The Japanese Society of Gastroenterology^12^. VDZ treatment for the induction of remission was defined as VDZ started for active UC (Lichtiger index^13^ was ≥ 5).

### Assessment of clinical efficacy

Clinical response at week 22 was assessed using the Lichtiger index^13^. Clinical remission was defined as a Lichtiger index of 4 or lower. Subjects who terminated VDZ treatment (switching to other medications) or needed surgery because of insufficient control of UC disease activity before week 22 were regarded as not achieving clinical remission at week 22.

### Machine learning and prediction tools

To investigate clinical features related to SFCR during VDZ treatment at week 22, the data of 49 clinical features at week 0 were obtained from the Kyorin medical record system for patients in Cohort 1. The examined features included sex, age, height, body weight, body mass index (BMI), disease duration, disease type (inflammation distribution), treatment history for UC, clinical activity, endoscopic activity, and 25 blood test items (Table 1). The blood test was performed on the day of the first VDZ dose. Colonoscopy performed within 3 months before starting VDZ therapy was employed to obtain the baseline endoscopic findings. Categorical data were replaced with dummy variables. Missing values were imputed with the average value and the mode value for numerical data and categorical data, respectively. The data of patients in Cohort 2 were similarly collected from the Toho Sakura Medical Center medical record system. The standardized values of Cohort 1 were used for RF. RF was employed to develop a high-accuracy prediction model and identify which feature contributed to the prediction in the present study. RF is an ensemble technique using decision trees. In training, the RF algorithm creates multiple trees, and each tree is trained on the bootstrapped samples of the training data. Since the number of patients was limited in this study, RF was initialized using random values, and the training of RF was repeated 50 times. The contribution of each feature (49 clinical features, Table 1) to SFCR at week 22 was obtained by calculating the average value. When training the RF, the hyperparameters (number of trees and maximum depth of the tree) were automatically optimized via grid search and cross-validation. Grid search is a method for obtaining optimal hyperparameters in an algorithm. This performs a complete search over a given subset of the hyperparameter space of the training algorithm. The best hyperparameters are estimated according to the evaluation score of the validation data. Cross-validation is a resampling procedure for evaluating machine-learning models on a limited data sample. The general procedure is as follows: (1) split the dataset into k groups; (2) for each group, (i) select a group as a validation dataset, (ii) use the remaining groups (“k − 1” groups) as a training dataset, and (iii) fit a model on the training set and evaluate it on the validation set; and (3) calculate an average of k evaluation score. The final prediction result is obtained from the mode of predictions obtained from individual decision trees. The feature importance is determined according to the extent a decision tree node using each feature can reduce impurity across all trees in the forest. Next, logistic regression was used to develop a prediction tool in this study. Logistic regression is a classification algorithm for assigning each observation to a discrete set of classes. We inputted eight clinical features at week 0 that were selected as features with high contributions based on RF findings to predict the achievement/no achievement of SFCR at week 22. Logistic regression finally outputted the probability of which an observation vector belongs to a particular class using the logistic sigmoid function. The prediction accuracy of the model was assessed using the data of Cohort 2. We performed the machine learning in python and used the scikit-learn package.

**Table 1:** Baseline clinical features employed for machine learning.

Background	Clinical activity of UC	Endoscopic activity of UC	Complete blood count	Blood chemistry
Sex (M/F)	Lichtiger index	Mayo endoscopic subscore	Red blood cell (× 10^4^/µL)	BUN (mg/dL)
Age (years old)	Partial Mayo score	UCEIS	Hemoglobin (g/dL)	Creatinine (mg/dL)
Height (cm)		UCEIS-V	Hematocrit (%)	eGFR (mL/min)
Body weight (kg)		UCEIS-E	MCV (fL)	Total bilirubin (mg/dL)
Body mass index		UCEIS-B	MCH (pg)	AST (IU/L)
UC disease duration (years)			MCHC (g/dL)	ALT (IU/L)
UC disease type			White blood cell (× 10^3^/µL)	GGT (IU/L)
**Treatment history for UC**			Neutrophil (%)	Total protein (g/dL)
5-ASA			Eosinophil (%)	Albumin (g/dL)
Azathioprine			Basophil (%)	Globulin (g/dL)
Prednisolone			Monocyte (%)	Total cholesterol (mg/dL)
Anti TNF-alpha agent			Lymphocyte (%)	CRP (mg/dL)
Tofacitinib			Platelet (× 10^4^/µL)	
Tacrolimus				
GMA				
**Concomitant treatment for UC**				
5-ASA				
Azathioprine				
Prednisolone				

*UC* ulcerative colitis, *5-ASA* 5-aminosalicylic acid, *TNF* tumor necrosis factor, *GMA* granulocyte and monocyte apheresis, *UCEIS* ulcerative colitis endoscopic index of severity, *MCV* mean corpuscular volume, *MCH* mean corpuscular hemoglobin, *MCHC*, mean corpuscular hemoglobin concentration, *BUN* blood urea nitrogen, *eGFR* estimated glomerular filtration rate, *AST* aspartate aminotransferase, *ALT* alanine aminotransferase, *GGT* gamma-glutamyl transpeptidase, *CRP* C-reactive protein.

### Ethical considerations

This study was conducted in accordance with the guidelines of the Declaration of Helsinki. This study was approved by the Institutional Ethics Committees of Kyorin University School of Medicine (Approval Number 1364) and Toho University Sakura Medical Center (Approval Number S20071). Informed consent was obtained from subjects (also from a parent when a patient was younger than 18 years) prior to the study.

## Results

### Training dataset

The clinical demographics of the 34 patients in Cohort 1 at baseline are presented in Table 2. The cohort consisted of 25 men and 9 women with a median age of 37 years (range, 17–92 years). The median disease duration was 5.0 years (range, 0.1–31.0 years). The disease diagnosis was total colitis in 28 patients and left-sided colitis in six patients. Thirty-three patients (97.1%) had been treated with 5-ASA before starting VDZ, seven of whom stopped 5-ASA treatment because of intolerance. Thirteen patients (38.2%) had previously received AZA before VDZ, three of whom stopped AZA therapy because of adverse events. In total, 29 (85.3%), 12 (35.3%), 1 (2.9%), 3 (8.8%), and 3 (8.8%) patients had been treated with prednisolone (PSL), anti-TNFα agents, TOF, TAC, and granulocyte and monocyte apheresis (GMA), respectively, before starting VDZ. No patient stopped these treatments because of adverse events. When starting VDZ (week 0), 21 (61.8%), 8 (23.5%), and 8 (23.5%) patients were using 5-ASA, AZA, and PSL, respectively. The clinical disease activity at baseline was assessed using the Lichtiger index and partial Mayo (pMayo) score for all patients (Table 2). Colonoscopy was performed at baseline in 31 patients. Endoscopic disease activity was assessed using the Mayo endoscopic subscore (MES) and ulcerative colitis endoscopic index of severity (UCEIS) (Table 2). The results of 25 blood test items at week 0 are presented in Table 2. At week 22, among the 34 patients, 18 patients (52.9%; 12 males and 6 females) achieved SFCR with VDZ. No patient stopped VDZ because of adverse events.

**Table 2:** Clinical demographics of Cohort 1 (34 patients).

**Background**		**Complete blood count**^**#**^	
Sex (M/F)	25/9	Red blood cell (× 10^4^/µL) (n = 34)	422.4 ± 12.13
Age (years old) (median, range)	37 (17–92)	Hemoglobin (g/dL) (n = 34)	12.49 ± 0.3149
Height (cm)*^, #^	164.4 ± 1.6	Hematocrit (%) (n = 34)	37.69 ± 0.9129
Body weight (kg)*^,#^	54.4 ± 1.9	MCV (fL) (n = 34)	89.93 ± 1.191
Body mass index*^,#^ (mean, range)	20.1 ± 0.6	MCH (pg) (n = 34)	29.84 ± 0.4896
UC disease duration (years) (median, range)	5.0 (0.1–31.0)	MCHC (g/dL) (n = 34)	33.15 ± 0.1728
UC disease type (total colitis/left-sided colitis)	28/6	White blood cell (× 10^3^/µL) (n = 34)	7.431 ± 0.4484
**Treatment history for UC**		Neutrophil (%) (n = 32)	67.63 ± 1.625
5-ASA (+ /−)	33/1	Eosinophil (%) (n = 32)	3.258 ± 0.6002
Azathioprine (+ /−)	13/21	Basophil (%) (n = 32)	0.5636 ± 0.07951
Prednisolone (+ /−)	29/5	Monocyte (%) (n = 32)	8.976 ± 0.5657
Anti TNF-alpha agent (+ /−)	12/22	Lymphocyte (%) (n = 32)	19.52 ± 1.392
Tofacitinib (+ /−)	1/33	Platelet (× 10^4^/µL) (n = 34)	33.77 ± 1.652
Tacrolimus (+ /−)	3/31	**Blood chemistry**^**#**^	
Granulocyte and monocyte apheresis (+ /−)	3/31	BUN (mg/dL) (n = 31)	9.825 ± 0.8679
**Concomitant treatment for UC**		Creatinine (mg/dL) (n = 32)	0.7091 ± 0.02694
5-ASA (+ /−)	21/13	eGFR (mL/min) (n = 32)	98.73 ± 4.065
Azathioprine (+ /−)	8/26	Total bilirubin (mg/dL) (n = 32)	0.4188 ± 0.03491
Prednisolone (+ /−)	8/26	AST (IU/L) (n = 34)	18.44 ± 1.624
**Clinical activity of UC**		ALT (IU/L) (n = 34)	15.03 ± 2.051
Lichtiger index (median, range)	9 (5–16)	GGT (IU/L) (n = 30)	29.47 ± 5.878
Partial Mayo score (median, range)	6 (4–9)	Total protein (g/dL) (n = 34)	6.540 ± 0.1535
**Endoscopic activity of UC (n = 31)**		Albumin (g/dL) (n = 33)	3.447 ± 0.1301
Mayo endoscopic subscore (median, range)	2 (2–3)	Globulin (g/dL) (n = 33)	3.094 ± 0.08412
UCEIS	6 (3–9)	Total cholesterol (mg/dL) (n = 27)	147.3 ± 6.191
UCEIS-V	2 (1–3)	CRP (mg/dL) (n = 32)	1.780 ± 0.4263
UCEIS-E	2 (1–3)	Achievement of SFCR at week 22 (yes/no)	18/16
UCEIS-B	2 (0–3)		

*Height, body weight, and body mass index were not measured in one subject.

^#^Height, body weight, body mass index and the results of blood tests are described as the mean ± SEM.

### Test dataset

The clinical demographics of the 35 patients in Cohort 2 at baseline are presented in Table 3. This cohort included 22 men and 13 women with a median age of 42 years (range, 17–90 years). The median disease duration was 7.2 years (range, 0.6–38.0 years). The disease diagnosis was total colitis in 26 patients and left-sided colitis in nine patients. treatment history of 5-ASA, AZA, PSL, anti-TNFα agent, TOF, TAC, and GMA treatment before VDZ therapy was documented in 35 (100%), 16 (45.7%), 34 (97.1%), 20 (57.1%), 6 (17.1%), 9 (25.7%), and 13 patients (37.1%), respectively. No patient stopped treatment because of adverse events. When starting VDZ (week 0), 29 (82.9%), 4 (11.4%), and 11 (31.4%) patients were using 5-ASA, AZA, and PSL, respectively. The clinical disease activity at baseline was assessed using the Lichtiger index and pMayo score for all patients (Table 3). Colonoscopy was performed at baseline in 14 patients, and their endoscopic disease activity was assessed using MES and UCEIS (Table 3). SFCR at week 22 was achieved in 13 patients (37.1%; seven men and six women). No patient stopped VDZ because of adverse events.

**Table 3:** Clinical demographics of Cohort 2 (35 patients).

**Background**		**Complete blood count**^**#**^	
Sex (M/F)	22/13	Red blood cell (× 10^4^/µL) (n = 35)	436.6 ± 10.94
Age (years old) (median, range)	42 (17–90)	Hemoglobin (g/dL) (n = 35)	11.92 ± 0.3198
Height (cm)*^, #^ (mean, range)	165.0 ± 1.4	Hematocrit (%) (n = 35)	37.12 ± 0.8463
Body weight (kg)*^, #^ (mean, range)	57.7 ± 1.9	MCV (fL) (n = 35)	85.46 ± 1.230
Body mass index*^, #^ (mean, range)	21.0 ± 0.6	MCH (pg) (n = 35)	27.57 ± 0.5806
UC disease duration (years) (median, range)	7.2 (0.6–38.0)	MCHC (g/dL) (n = 35)	31.86 ± 0.2824
UC disease type (total colitis/left-sided colitis)	26/9	White blood cell (× 10^3^/µL) (n = 35)	8.980 ± 0.4852
**Treatment history for UC**		Neutrophil (%) (n = 35)	68.74 ± 2.414
5-ASA (+ /−)	35/0	Eosinophil (%) (n = 35)	2.411 ± 0.4785
Azathioprine (+ /−)	16/19	Basophil (%) (n = 35)	0.5057 ± 0.07658
Prednisolone (+ /−)	34/1	Monocyte (%) (n = 35)	5.783 ± 0.3977
Anti TNF-alpha agent (+ /−)	20/15	Lymphocyte (%) (n = 35)	22.13 ± 2.096
Tofacitinib (+ /−)	6/29	Platelet (× 10^4^/µL) (n = 35)	37.60 ± 1.796
Tacrolimus (+ /−)	9/26	**Blood chemistry**^**#**^	
Granulocyte and monocyte apheresis (+ /−)	13/22	BUN (mg/dL) (n = 35)	11.44 ± 0.6232
**Concomitant treatment for UC**		Creatinine (mg/dL) (n = 35)	0.7643 ± 0.02761
5-ASA (+ /−)	29/6	eGFR (mL/min) (n = 34)	82.91 ± 2.631
Azathioprine (+ /−)	4/31	Total bilirubin (mg/dL) (n = 35)	0.5171 ± 0.03678
Prednisolone (+ /−)	11/24	AST (IU/L) (n = 35)	16.71 ± 1.498
**Clinical activity of UC**		ALT (IU/L) (n = 35)	15.37 ± 2.743
Lichtiger index (median, range)	7 (5–12)	GGT (IU/L) (n = 28)	28.57 ± 7.053
Partial Mayo score (median, range)	5 (2–8)	Total protein (g/dL) (n = 35)	7.303 ± 0.1118
**Endoscopic activity of UC (n = 31)**		Albumin (g/dL) (n = 35)	3.689 ± 0.08419
Mayo endoscopic subscore (median, range)	2.5 (0–3)	Globulin (g/dL) (n = 35)	3.614 ± 0.07758
UCEIS	5.5 (0–7)	Total cholesterol (mg/dL) (n = 33)	177.3 ± 6.781
UCEIS-V	2 (0–2)	CRP (mg/dL) (n = 35)	1.091 ± 0.2146
UCEIS-E	2 (0–3)	Achievement of SFCR at week 22 (yes/no)	13/22
UCEIS-B	1.5 (0–3)		

*Height and body mass index were not measured in three subjects.

^#^The results of blood tests are described as the mean ± SEM.

### Development of prediction tool for vedolizumab efficacy

**Figure 1:**
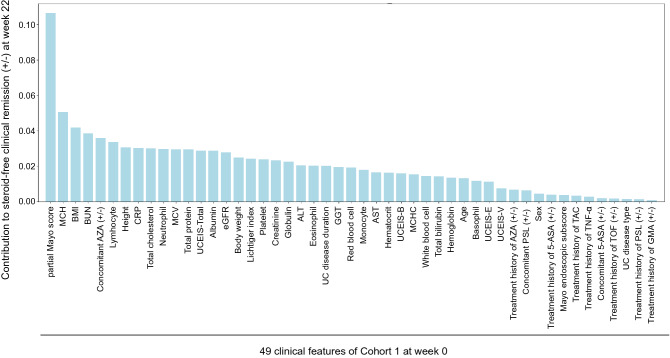
Contributions of 49 clinical features at week 0 to the likelihood of steroid-free clinical remission (SFCR) at week 22. The contribution of each baseline clinical feature to SFCR at week 22 was determined using the random forest algorithm. *MCH* mean corpuscular hemoglobin, *BMI* body mass index, *BUN* blood urea nitrogen, *AZA* azathioprine, *CRP* C-reactive protein, *MCV* mean corpuscular volume, *UCEIS* ulcerative colitis endoscopic index of severity, *eGFR* estimated glomerular filtration rate, *ALT* alanine amino transferase, *GGT* gamma-glutamyl transpeptidase, *MCHC* mean corpuscular hemoglobin concentration, *5-ASA* 5-aminosalicylic acid, *TAC* tacrolimus, *TNF* tumor necrosis factor, *TOF* tofacitinib, *PSL* prednisolone, *GMA* granulocyte and monocyte apheresis.

RF using the data of 49 clinical features at baseline for patients in Cohort 1 was performed, and the contribution of each factor to SFCR at week 22 was determined (Fig. 1). The 10 clinical features with the highest contribution were the pMayo score, mean corpuscular hemoglobin (MCH) concentration (pg), BMI, blood urea nitrogen (BUN) concentration (mg/dL), concomitant use of AZA (+ / −), lymphocyte (Lympho) fraction (%), height (cm), C-reactive protein (CRP) concentration (mg/dL), total cholesterol (TCho) concentration (mg/dL), and neutrophil fraction (%). These features were employed for logistic regression to develop a prediction model. The predictive accuracy of the logistic regression models (achievement of SFCR at week 22: y = 1, no achievement of SFCR at week 22: y = 0, threshold: y = 0.5) in Cohorts 1 and 2 is presented in Table 4. When the top 8 features (pMayo score, MCH, BMI, BUN, concomitant use of AZA, Lympho fraction, height, and CRP) were employed, the predictive accuracy was 100% in Cohort 1, versus 68.6% in Cohort 2. The equation of logistic regression using the features was as follows:

$$ {\text{y}} = 1/(1 + {\text{e}}{\,\hat\,}( - {\text{x}})). $$

$$ {\text{x}} = {\text{a}}_{0} \times \left[ {{\text{standardized}} - {\text{pMayo score}}} \right] + {\text{a}}_{{1}} \times \left[ {{\text{standardized}} - {\text{MCH}}} \right] + {\text{a}}_{{2}} \times \left[ {{\text{standardized}} - {\text{BMI}}} \right] + {\text{a}}_{{3}} \times \left[ {{\text{standardized}} - {\text{BUN}}} \right] + {\text{a}}_{{4}} \times \left[ {\text{concomitant use of AZA}} \right] + {\text{a}}_{{5}} \times \left[ {{\text{standardized}} - {\text{Lympho fraction}}} \right] + {\text{a}}_{{6}} \times \left[ {{\text{standardized}} - {\text{height}}} \right] + {\text{a}}_{{7}} \times \left[ {{\text{standardized}} - {\text{CRP}}} \right] \, - \, 0.{27955142}. $$

$$ {\text{a}}_{0} = - {2}.0{9616139}0{278958},{\text{ a}}_{{1}} = {1}.0{592561253594117},{\text{ a}}_{{2}} = - 0.{34465735}0{8632}0{3}0{4},{\text{ a}}_{{3}} = {2}.{7}0{5485}0{91}0{49323},{\text{ a}}_{{4}} = {6}.{718131278}0{58346},{\text{ a}}_{{5}} = {1}.{5638386797677}0{9},{\text{ a}}_{{6}} = - {2}.{523}0{13372748}0{39},{\text{ a}}_{{7}} = {1}.{96491}0{7396733663}. $$

$$ \begin{gathered} {\text{Standardized}} - {\text{pMayo score}} = \left( {{\text{pMayo score }} - { 6}.{235294118}} \right)/{1}.{284725275}. \hfill \\ {\text{Standardized}} - {\text{MCH}} = \left( {{\text{MCH }} - { 29}.{84411765}} \right)/{2}.{896365997}. \hfill \\ {\text{Standardized}} - {\text{BMI}} = \left( {{\text{BMI }} - { 2}0.0{7734956}} \right)/{3}.{472}0{63638}. \hfill \\ {\text{Standardized}} - {\text{BUN}} = \left( {{\text{BUN }} - { 9}.{8258}0{6452}} \right)/{4}.{9}0{95672}0{7}. \hfill \\ \end{gathered} $$

$$ \begin{gathered} {\text{Concomitant use of AZA}} = 0 \, \left( {{\text{No}}} \right){\text{ or 1 }}\left( {{\text{YES}}} \right). \hfill \\ {\text{Standardized}} - {\text{Lympho fraction}} = \left( {{\text{Lympho fraction }} - { 19}.{515625}} \right)/{7}.{9973}0{85}0{7}{\text{.}} \hfill \\ {\text{Standardized}} - {\text{height}} = \left( {{\text{height }} - { 164}.{39}0{9}0{91}} \right)/{9}.0{46524986}{\text{.}} \hfill \\ {\text{Standardized}} - {\text{CRP}} = \left( {{\text{CRP }} - { 1}.{779375}} \right)/{2}.{448675583}{\text{.}} \hfill \\ \end{gathered} $$

**Table 4:** Predictive accuracy of logistic regression models for steroid-free clinical remission at week 22 comprising the top 10 contributing clinical features.

Clinical features employed for the logistic regression model	Prediction accuracy in Cohort 1 (%)	Prediction accuracy in Cohort 2 (%)
Partial Mayo score	82.4	42.9
Partial Mayo score, MCH	76.5	60.0
Partial Mayo score, MCH, BMI	82.4	62.9
Partial Mayo score, MCH, BMI, BUN	76.5	60.0
Partial Mayo score, MCH, BMI, BUN, Concomitant AZA (+ /−)	82.4	60.0
Partial Mayo score, MCH, BMI, BUN, Concomitant AZA (+ /−), Lymphocyte	82.4	60.0
Partial Mayo score, MCH, BMI, BUN, Concomitant AZA (+ /−), Lymphocyte, Height	88.2	62.9
Partial Mayo score, MCH, BMI, BUN, Concomitant AZA (+ /−), Lymphocyte, Height, CRP	100.0	68.6
Partial Mayo score, MCH, BMI, BUN, Concomitant AZA (+ /−), Lymphocyte, Height, CRP, Total Cholesterol	100.0	68.6
Partial Mayo score, MCH, BMI, BUN, Concomitant AZA (+ /−), Lymphocyte, Height, CRP, Total Cholesterol, Neutrophil	100.0	65.7

*MCH* mean corpuscular hemoglobin, *BMI* body mass index, *BUN* blood urea nitrogen, *AZA* azathioprine, *CRP* C-reactive protein.

The calculated value of y and the accuracy of the prediction in each patient in Cohorts 1 and 2 are presented in Supplemental Table 1. In Cohort 2, the positive predictive value (achievement of SFCR) and negative predictive value (NPV; no achievement of SFCR) were 54.5% and 92.3%, respectively (Table 5).

**Table 5:** Predictive ability of the model steroid-free clinical remission at week 22 in Cohort 2.

	Steroid-free clinical remission at week 22	
	( +)	(−)
Prediction of ( +)	12	10
Prediction of (−)	1	12

Positive predictive value: 54.5%.

Negative predictive value: 92.3%.

Accuracy: 68.6%.

## Discussion

In the present study, we analyzed 49 clinical features at week 0 using real-world data with the RF algorithm and determined the contribution of each clinical feature to the achievement of SFCR after 22 weeks of VDZ therapy. It is an advantage of RF that we could investigate the contribution of these various clinical features in our cohorts despite the limited the number of subjects. Generally, it is challenging to assess a large number of features in detail using statistical methodology, such as univariate and multivariate analyses, which require a huge number of subjects. In addition, we believe that we need to interpret the “*p*-value” in statistical analyses carefully, although we acknowledge statistical significance provides scientific insights. Some factors without statistical significance may potentially contribute to the outcome. Assessing the contribution of factors comprehensively with RF could be a promising approach for identifying predictors, particularly in a complex situation in which various factors can be involved as such SFCR after VDZ treatment. Logistic regression was employed in this study to develop a prediction tool with clinical features using the eight largest contributors; pMayo score, MCH (pg), BMI, BUN (mg/dL), concomitant use of AZA, Lympho fraction (%), height (cm), and CRP (mg/dL). Our model revealed a high NPV (92.3%) for SFCR at week 22. This finding suggests that it would be better to consider other options if our model predicts VDZ will be ineffective for an individual patient. In the logistic regression model, the coefficient of each factor indicates if a factor is positively or negatively associated with the outcome. Our logistic regression model illustrated that a lower pMayo score, higher MCH concentration, lower BMI, higher BUN concentration, concomitant use of AZA, higher Lympho fraction percentage, shorter height, and higher CRP concentration at week 0 were favorable for SFCR at week 22. We believe that interpreting the machine-learning results from medical and physiological viewpoints is crucial for considering the clinical significance of the model, and it could provide an opportunity to improve clinical practice.

A lower pMayo score indicates less clinical disease activity^14^. Higher MCH levels suggest that bleeding attributable to UC and iron, vitamin B_12_, or folic acid deficiency are less severe. Since no patient had overt renal dysfunction in our cohorts, higher BUN levels are believed to reflect the intake of sources of nitrogen, i.e., patients’ dietary intake, particularly amino acids. Taken together, these factors imply that less disease activity and a better general and nutritional status are favorable for SFCR during VDZ therapy. In the present study, TCho (mg/dL) was one of the nine strongest contributors in RF, and when we included this feature in the logistic regression model, its coefficient was positive. Because TCho levels are decreased in response to malnutrition induced by active inflammation, this finding also suggests a better nutritional condition is positively related to VDZ efficacy. Barré et al. reviewed several reports on the predictors of VDZ treatment for UC and noted that severe disease activity at induction is a negative predictor^6^. Dulai et al. developed a tool to predict the response to VDZ including baseline moderate activity on endoscopy and albumin levels as positive predictors^7^. Our findings and interpretations of the pMayo score, MCH level, and BUN level appear compatible with these previous studies. Interestingly, lower BMI and shorter height were included as positive predictors in our prediction model. We speculate that these factors suggest a high VDZ concentration in the body because the dose was fixed as 300 mg/injection. In the GEMINI I study, a positive correlation was observed between VDZ serum concentrations and clinical response^15^. Samaan et al. reported that VDZ dose intensification was effective in patients with IBD with a suboptimal treatment response^16^. In a review by Barré et al., a low trough level of VDZ is cited as a negative predictor^6^. Together with these reports and our findings, we speculate that adjusting the dose of VDZ depending on BMI could increase its efficacy. Meanwhile, caution may be needed when applying our prediction tool to patients with overt emaciation that far exceeds the range observed in the training dataset. It is noteworthy that the concomitant use of AZA was detected as a positive predictor, and the absolute value of its coefficient was the largest in our model; i.e., concomitant AZA use has a larger impact on SFCR at week 22 than the other features. Whereas the benefit of the combination of an immunomodulator and VDZ over VDZ monotherapy has not been established, our machine-learning approach identified the potentially beneficial effect of concomitant AZA use. We believe that the results for BMI/height and concomitant AZA use raise an important clinical question concerning the optimization of VDZ treatment for UC. Meanwhile, our finding that a higher Lympho fraction was related to SFCR during VDZ treatment suggests that VDZ responders could comprise a subgroup of UC with a specific pathophysiology. VDZ is a humanized monoclonal antibody directed toward α_4_β_7_ integrin. α_4_β_7_ integrin is expressed on the surface of lymphocytes, and it interacts with mucosal addressin cell adhesion molecule-1 (MAdCAM-1), which leads to the migration of lymphocytes to the intestine^17^. Based on this specific mechanism and our finding, we speculate that there could be a “lymphocyte-dominant” subgroup of UC, and VDZ exerts particularly efficacy in such patients. The machine-learning approach would be useful for developing a prediction tool and obtaining clues for characterizing UC pathophysiology and subgrouping patients. Our model indicated that higher CRP levels were related to SFCR at week 22. This finding is incompatible with a previous report^6^, and it appears inconsistent with the favorability of a lower pMayo score. Among subjects with and without SFCR at week 22 in the training dataset, the mean and standard error of the mean (SEM) of CRP levels were 1.566 ± 0.6187 mg/dL and 2.054 ± 0.6328 mg/dL, respectively (*p* = 0.0532, Mann–Whitney *U* test). However, four subjects who achieved SFCR had a high CRP level (8.37 mg/dL, 6.43 mg/dL, 6.34 mg/dL, and 2.25 mg/dL, respectively), whereas the level was 0.02–1.88 mg/dL in the other patients who achieved SFCR (the normal CRP level is ≤ 0.14 mg/dl). Given that CRP levels were not high overall in Cohort 1, the results of these four patients might affect the decision of the machine-learning algorithm.

We consider three future directions of the machine-learning approach for UC clinical data: (1) aiming for higher prediction accuracy, (2) developing prediction tools for various medications, and (3) searching for factors potentially involved in UC pathophysiology. Regarding (1), this study was limited by its small size. Larger training and test cohorts are needed to improve the prediction model and its accuracy. Additionally, it will be interesting to test other machine-learning methodologies, such as k-NN and support vector machine, and determine if those approaches can generate a better model. Point (2) is crucial for personalized medicine for UC. That is, if we have a prediction tool for each therapeutic intervention, we can run the multiple tools at baseline and determine which intervention is most suitable for individual patients. For instance, whereas Dulai et al. developed a prediction model for VDZ efficacy in patients with Crohn’s disease (CD)^18^, Alric et al. observed that the model could not predict the efficacy of UST in patients with CD^19^. Several cutting-edge studies are exploring the predictors of VDZ efficacy in patients with IBD. Ananthakrishnan et al. reported that the functional profile of the gut microbiome can be a predictor of VDZ efficacy at week 14 in patients with IBD^20^. Rath et al. analyzed peripheral blood and colonic biopsy samples for CD4^+^ T cell subpopulations, cytokine production, and mRNA and protein expression including the α_4_β_7_ integrin and MAdCAM-1 to investigate factors associated with VDZ efficacy in patients with IBD and revealed a significant difference in genetic signatures at baseline between subjects with and without clinical remission at week 14^21^. Verstockt et al. employed machine-learning methods and reported that the expression of four genes in colon tissue could be predictive of VDZ efficacy in patients with IBD^22^. Gazouli et al. analyzed the mucosal expression of immunological and inflammatory genes using a machine-learning algorithm and demonstrated that the response to VDZ in patients with UC is associated with mucosal gene expression profiles at baseline^23^. Although these findings are interesting, at present, they cannot be feasibly examined in a clinical setting. We believe it is advantageous to analyze common clinical features that can be obtained in a clinical setting to allow application of the predictors and prediction models in daily practice. Regarding point (3), adding experimental factors to the metadata for machine learning may provide opportunities to investigate novel factors associated with outcomes and understand the underlying pathological features of UC. Previous studies demonstrated that mucosal gene expression profiles are related to the treatment response of patients with UC^24,25^. Kim et al. reported that mucosal eosinophilia is a predictor of VDZ efficacy in patients with IBD^26^. These findings suggest the possibility that more factors that contribute to the clinical outcome have not been examined in daily practice. Analyzing various hypothetical predictors (e.g., cytokine levels, gene expression, histological characteristics) together with machine-learning approaches would provide insights into the contribution of each factor and facilitate the discovery of the characteristics of UC subgroups. In conclusion, with machine learning, we determined the contribution of clinical features at week 0 to the achievement of SFCR in patients who received VDZ for UC at week 22 and developed a prediction model. The predictive accuracy was confirmed in a separate cohort. The concept and findings in this study will promote personalized medicine in UC, and they could possibly be extrapolated to other medications and diseases.

## Supplementary Information

Supplementary Table S1.

## Data Availability

The data underlying this article will be shared by the corresponding author upon reasonable request.

## Abbreviations

5-ASA: 5-Aminosalicylic acid
AZA: Azathioprine
BMI: Body mass index
BUN: Blood urea nitrogen
CD: Crohn’s disease
CRP: C-reactive protein
GMA: Granulocyte and monocyte apheresis
JAK: Janus kinase
Lympho: Lymphocyte
MadCAM-1: Mucosal addressin cell adhesion molecule-1
MCH: Mean corpuscular hemoglobin
MES: Mayo endoscopic subscore
NPV: Negative predictive value
pMayo score: Partial Mayo score
PPV: Positive predictive value
PSL: Prednisolone
RF: Random forest
SFCR: Steroid-free clinical remission
TAC: Tacrolimus
TCho: Total cholesterol
TNF: Tumor necrosis factor
TOF: Tofacitinib
UC: Ulcerative colitis
UCEIS: Ulcerative colitis endoscopic index of severity
UST: Ustekinumab
VDZ: Vedolizumab
